# Developing and Integrating Virtual Reality Courses in Medical Education: Tutorial and Implementation Guideline Informed by Best Practices From the National Project “medical tr.AI.ning”

**DOI:** 10.2196/80976

**Published:** 2026-05-01

**Authors:** Marvin Mergen, Anna Junga, Henriette Schulze, Philipp Bozdere, Pascal Kockwelp, Leon Pielage, Corbin Sassen, Tina Glückselig, Michael Schmitz, Kathrin Ungru, Norbert Graf, Benjamin Risse, Marcel Meyerheim

**Affiliations:** 1 Department of Pediatric Oncology and Hematology Faculty of Medicine Saarland University Homburg Germany; 2 Institute for Education and Student Affairs University of Münster Münster Germany; 3 Institute for Society and Digital Media, Münster School of Design FH Münster Münster Germany; 4 Institute for Geoinformatics University of Münster Münster Germany; 5 xm:lab - experimental media lab Hochschule der Bildenden Künste Saar Saarbrücken Germany; 6 Department of Electrical Engineering and Computer Science Laboratory of Visual Computing FH Münster Münster Germany

**Keywords:** virtual reality, project management, artificial intelligence, interdisciplinary collaboration, tutorial

## Abstract

The increasing adoption of virtual reality (VR) in medical education offers substantial opportunities for immersive, practice-oriented training that complements traditional teaching methods. In particular, VR enables repeated, risk-free exposure to complex clinical scenarios and supports the development of clinical reasoning, communication skills, and procedural competence. However, implementing VR-based courses remains challenging due to high development costs, technical complexity, and the need for close interdisciplinary collaboration. This tutorial presents key insights and best practices from the medical tr.AI.ning project, a 3-year interdisciplinary initiative funded by the German Federal Ministry of Education and Research. The project’s objective was to develop an artificial intelligence (AI)-supported, VR-based training platform that allows medical students to practice clinical decision-making in immersive, interactive scenarios. The paper is structured as a tutorial and offers recommendations for planning, developing, and integrating VR courses into medical curricula. Each recommendation is illustrated with concrete examples from our project, serving as a practical blueprint to guide educators and developers in applying these guidelines in their own contexts. Successful implementation of a VR project in medical education requires strategic planning and collaboration, starting with a thorough identification of curricular gaps that VR can address and a clear justification of its added educational value. An interdisciplinary consortium that combines expertise from medical didactics experts, computer science, and design is essential to ensure the development of high-quality, pedagogically sound simulations and intuitive user interfaces. Key factors for success include defining specific learning objectives aligned with competency-based frameworks; iterative development with continuous feedback from medical experts, educators, and students; and structured pilot testing with systematic collection of quantitative and qualitative data to assess usability, immersion, and learning outcomes. Early engagement and walkthroughs with end users help identify practical challenges and inform iterative improvements. A dedicated authoring tool within the project allows medical teachers to create and adapt VR scenarios without prior technical experience, supporting the scalability and sustainability of the approach. Effective project management frameworks facilitate collaboration, clear task allocation, and adaptive progress throughout development. Additionally, considerations for hardware selection, technical infrastructure, and sustainable dissemination strategies, including open-access publications, project websites, and professional networking, are crucial to ensure long-term viability and broad adoption across institutions. By combining a tutorial format with practical, step-by-step recommendations, this article provides a comprehensive guide for educators and developers on implementing immersive, AI-supported VR courses to enhance medical education. It highlights key lessons learned in interdisciplinary collaboration, iterative testing, systematic evaluation, and alignment with educational objectives, thereby facilitating the effective, evidence-based, and sustainable integration of VR into medical curricula across diverse institutions.

## Introduction

Medical education is increasingly adopting digital learning methods, driven by advancements in computer science, particularly in artificial intelligence (AI). As part of this shift, virtual reality (VR) is emerging as a valuable training tool for both aspiring and practicing health care professionals [1]. Initially, VR was used predominantly for training surgical interventions and procedures [1-3], but its applications are expanding rapidly across a range of other use cases. The motivation of medical educators to invest in and learn to use this new technology derives mainly from distinct advantages, such as the ability to repeat medical procedures in a safe, immersive environment as often as needed, without risk to real patients or users themselves [1,3]. At the same time, VR still encounters certain challenges, including technical limitations in terms of haptic feedback [4,5] and issues related to cybersickness [6,7]. However, the growing interest in using VR for medical education is driving ongoing innovations that may help overcome these current limitations. Additionally, VR’s expanding role suggests potential for extended didactic approaches, further supporting its integration into curricula.

This paper is intentionally written as a tutorial informed by the 3-year, multiinstitutional project medical tr.AI.ning [8] (Germany; project start: December 2021). It synthesizes practical implementation lessons (planning, development, integration, and sustainability) rather than reporting a controlled effectiveness trial. While we refer to evaluation activities conducted during the project (eg, pilot courses and feasibility studies), the purpose of these activities in this tutorial is to inform implementation decisions, not to claim that VR is superior to other teaching formats.

### How to Use This Tutorial

The guidance is organized into 4 phases that readers can follow sequentially or consult as needed:

- Phase 1: How to start (needs assessment, consortium setup, goals, resources).
- Phase 2: How to organize (communication, project management, infrastructure).
- Phase 3: How to build (learning design, technical foundation, AI options, interdisciplinary workflows).
- Phase 4: How to integrate (iterative testing, curricular embedding, dissemination, sustainability).

**Figure 1:**
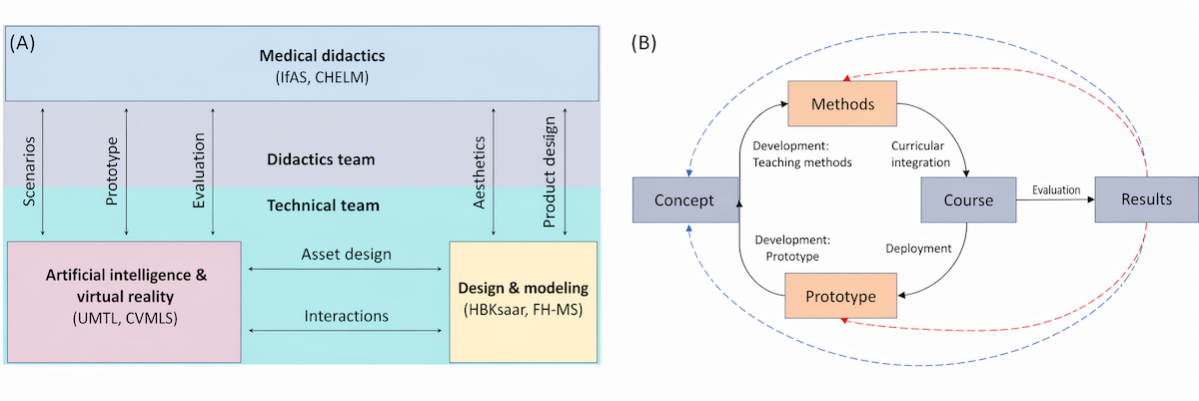
(A) Consortium structure and broadly defined responsibilities within the medical tr.AI.ning project; (B) Prototype life cycles. CHELM: Coordination Center Homburg for Education & Learning in Medicine; CVMLS: Computer Vision & Machine Learning Systems; FHMS: University of Applied Sciences Münster; HBKsaar: Hochschule der Bildenden Künste Saar; IfAS: Institut für Ausbildung und Studienangelegenheiten; UMTL: Ubiquitous Media Technologies Lab.

The medical tr.AI.ning project’s primary goal is to create an AI-supported, VR-based training platform that enables medical students to practice clinical decision-making in customizable, immersive virtual scenarios. Additionally, a key project component is the development of an authoring tool that provides medical teachers with a visual interface, allowing them to easily create and adapt new training scenarios tailored to specific educational needs. The project draws on contributions from the University of Münster (Institut für Ausbildung und Studienangelegenheiten [IfAS] and Computer Vision & Machine Learning Systems [CVMLS]), Saarland University (Coordination Center Homburg for Education & Learning in Medicine [CHELM] and Ubiquitous Media Technologies Lab [UMTL]), the University of Applied Sciences Münster (FHMS; Department of Electrical Engineering and Computer Science [ETI], Münster School of Design [MSD], and Institut Gesellschaft und Digitales [GUD]), and the Saarbrücken Academy of Fine Arts (xm:lab and Hochschule der Bildenden Künste Saar [HBKsaar]). Responsibilities and competencies are broadly divided into 2 core teams (see Figure 1). The medical didactics team (IfAS and CHELM) is responsible for developing teaching methods and strategies, providing the medical foundation necessary for programming and designing scenarios in collaboration with medical specialists, and deploying the developed content into medical curricula. The design and technical team (UMTL, CVMLS, FHMS, xm:lab, and HBKsaar) handles platform implementation, including design, modeling, animations, and the creation of a unified interaction concept for VR and the authoring tool. While tasks are clearly defined, close interdisciplinary collaboration is essential throughout the project life cycle.

In this paper, we synthesize key insights from this 3-year project to enhance transparency in university-led research initiatives and to derive structured, practice-oriented recommendations.

Existing publications on VR in medical education predominantly focus on single educational interventions, technical feasibility, usability, or learning outcomes, often within narrowly defined curricular contexts [9-11]. While several reviews and framework papers discuss general design considerations for immersive learning, comprehensive end-to-end guidance on how to systematically plan, implement, integrate, and sustain VR courses within real-world medical curricula remains scarce [1,12,13].

In particular, operational aspects such as consortium building, project management structures, interdisciplinary coordination, and long-term curricular integration are rarely addressed in a consolidated manner. This tutorial addresses this gap by providing a step-by-step, implementation-oriented guideline informed by a national, multiinstitutional project. Drawing on 3 years of iterative development, deployment, and evaluation across multiple medical faculties, this paper offers practical, reproducible guidance covering the full life cycle of VR course development—from needs assessment and team setup to technical implementation, curricular integration, and dissemination. These recommendations are grounded in practical experience and collaborative processes across multiple institutions.

Methodologically, the guidance is informed by structured workshops with medical educators, iterative usability testing, pilot studies with students, and formative and summative evaluations conducted throughout the project.

In this tutorial, we present general recommendations for developing and integrating VR courses in medical education, and we complement each recommendation with concrete examples from the medical tr.AI.ning project. These examples serve as a practical blueprint, illustrating how the guidelines can be applied in a real-world VR development context.

Feedback from medical experts, teachers, and learners was systematically incorporated into successive project phases and directly shaped the procedural steps outlined in this article. The resulting tutorial, therefore, reflects practice-based, empirically informed recommendations, providing both conceptual guidance and operational examples for future implementations.

### Phase 1: How to Start

#### Overview

This phase focuses on the initial conceptual stage of VR course development in medical education. It outlines how to identify educational needs, define realistic project goals, and establish the interdisciplinary foundations required for a successful VR project.

#### Defining Gap: Identifying Gaps in Medical Education That Benefit From VR

Identifying curricular gaps before integrating VR into medical education is crucial to ensure that the technology is applied strategically, targeting specific deficiencies in the current curriculum that are difficult or impossible to address with traditional learning methods. In practice, we recommend starting with a detailed review of existing learning objectives and mapping them to potential VR scenarios. This process involves defining a clear rationale for using VR, ensuring that it is selected for its distinct advantages over other teaching methods rather than being adopted purely for novelty.

VR offers immersive, interactive experiences that can simulate complex medical scenarios, such as high-risk procedures or examinations involving intimate areas, which we specifically addressed in the medical tr.AI.ning project. These scenarios are ideal candidates for VR, as they allow students to gain essential skills in a controlled, risk-free environment, which would be difficult or impossible to recreate with traditional methods such as lectures, manikins, real-life settings, or other forms of standardized simulations [14].

In addition, the customizable nature of VR simulations allows users to edit existing training scenarios or create new ones to align with various teaching specializations. This flexibility was a key reason for developing a dedicated authoring tool in our project.

Once the rationale is established, VR should be applied in a well-considered manner that aligns with overarching educational goals. The technology should enhance the learning experience rather than merely replace existing methods without adding benefits. Given that VR requires significant resources for development and implementation, its use must be justified by specific educational needs. The goal is not to replace patient contact or training with actors or simulated patients, but to supplement the curriculum with additional learning scenarios that fill existing educational gaps.

In the medical tr.AI.ning project, we therefore focused on the performance of a whole-body skin cancer screening, a procedure that is challenging for many medical students to practice in real-life settings. In particular, the examination of intimate body areas may be associated with embarrassment for both students and patients, which can limit opportunities for repeated, hands-on training. In addition, VR allows students to repeatedly practice the direct visual differentiation of potentially malignant lesions from benign skin findings under standardized conditions [14]. In real clinical settings, the availability of suitable patients (eg, with melanoma) is neither predictable nor equally distributed across teaching sessions. By embedding representative cases in VR, all students can be exposed to the same educational content and have equal opportunities to practice the required examination and decision-making steps.

#### Integrating AI

While the use of VR itself offers many benefits, additional value can be provided by AI technology. Real-time speech recognition and synthesis enable more natural, fluid conversations and eliminate the need for students to navigate predefined questions and commands, which might compromise the effectiveness of training in clinical decision-making. AI can enable dynamic, context-aware interactions with virtual agents, supporting adaptive, natural language responses that reflect realistic clinical dialogues [15]. This capability mirrors the unpredictability of real-world scenarios while providing a safe environment for students to practice and refine critical soft skills, such as empathy, active listening, and effective communication.

Furthermore, AI can be used to autonomously generate data protection-compliant clinical images [16]. This not only substantially increases the quantity and diversity of clinical images and their placement within the course, but also enhances the challenge for students to make accurate decisions.

#### Building the Consortium

##### Identifying and Recruiting Team Members (Medical Professionals/Didactics, IT Experts, 3D Designers, and Interaction Designers)

The success of an educational VR project heavily relies on assembling a carefully selected, multidisciplinary consortium. Securing expertise in medical didactics, computer science, and design is crucial to developing a comprehensive and impactful learning environment. When forming such a consortium, it is important to choose members with proven expertise in these domains, a strong record of collaborative work, and a shared commitment to educational innovation. Additionally, expertise in AI can substantially enhance the capabilities and outcomes of VR development. It is also important to define how medical specialists will be involved in the development and evaluation process. By ensuring these competencies are well represented, the project establishes a robust foundation for success.

Budget constraints may necessitate adjustments in project setup, such as reducing staffing levels. This can be addressed by focusing on specific goals and scaling back staffing in disciplines with lower workloads. To compensate, leveraging tools, libraries, and off-the-shelf solutions—such as standard user interface (UI) elements, 3D asset libraries, and VR world builders—can streamline development processes. While it is often inadvisable to entirely forgo a discipline, maintaining at least one senior team member to oversee and guide the effective use of these tools helps ensure quality and consistency.

Furthermore, the rapid evolution of generative AI offers new opportunities for innovative workflows in VR development. This advancement has the potential to substantially transform existing processes. Team members should stay actively informed about advancements in AI to identify and effectively integrate these developments into the project.

##### Defining Roles and Responsibilities

The inherent size and interdisciplinarity of a consortium developing VR simulations for medical education require both a project manager and a project coordinator. The project coordinator fulfills a support role, primarily focusing on administrative tasks such as scheduling, reporting, and coordinating communication among team members. The project coordinator ensures that day-to-day activities align with the project’s objectives. By contrast, the project manager holds a leadership role, overseeing the entire project life cycle, including planning, resource allocation, risk management, and strategic decision-making. While the project manager adopts a high-level perspective and makes key decisions, the project coordinator focuses on operational aspects to ensure smooth execution.

Additionally, responsibilities within the teams at each location should be coordinated, ideally through team leaders who work closely on the respective topics.

From our experience, clearly defining responsibilities is especially important in interdisciplinary teams. Splitting the coordination role into technical and didactical (ie, nontechnical) components ensures that all needs and project goals are addressed. These coordinators must maintain constant communication and collaboratively address challenges, escalating issues to the project manager when necessary.

The technical coordinator is responsible for ensuring the technical implementation of the VR project, including the integration of hardware, software, and additional modules such as an authoring tool. This role involves monitoring the build process; diagnosing and resolving technical issues; managing updates and merge requests (ie, developers’ proposals to integrate source code changes); and maintaining performance and compatibility standards for the VR simulations.

The didactical coordinator ensures that the educational content and learning objectives are correctly integrated into the VR simulations. Collaborating closely with educators and medical experts, they align the simulations with current medical curricula, such as the NKLM 2.0 [17], and broader educational frameworks such as competency-based medical education. This role ensures that the simulations are pedagogically sound and meet the intended educational outcomes.

To streamline administrative tasks, an overall coordinator position can be beneficial. In our project, administrative responsibilities were also managed by the didactical coordinator.

Coordinating the technical aspects and workflows of such a multidisciplinary project across multiple sites (in our case, UMTL, CVMLS, FHMS, HBKsaar) is complex. In the medical tr.AI.ning project, the role of a central technical coordinator, as described above, was not planned in the granted project outline. Instead, each site operated under the leadership of a team leader who managed an interdisciplinary team and closely collaborated with the other sites. However, the absence of a central technical coordinator hindered cross-site collaboration and reduced overall project efficiency. It is recommended to establish a central technical coordinator to ensure efficient collaboration between sites and to guarantee the quality of the technical implementation, thereby achieving project goals more effectively. Table 1 suggests the distribution of roles and responsibilities as a RACI (responsible, accountable, consulted, and informed) matrix.

**Table 1:** The RACI^a^ matrix used to clarify responsibilities in the multisite virtual reality development consortium (medical tr.AI.ning, Germany; 2021-2024). Roles include project manager, didactic lead, technical lead, medical experts, and student assistants involved in course delivery and evaluation^b^.

Components	Project manager	Didactic lead	Technical lead	Medical expert	Student assistants
Needs assessment	A	R	C	R	I
Learning design	C	A/R	C	R	I
Virtual reality development	I	C	A/R	C	R
Technical testing	I	C	A/R	C	R
Curricular integration	R	A	C	R	I
Evaluation	R	A	C	C	R

^a^RACI: responsible, accountable, consulted, and informed.

^b^We recommend reviewing the RACI at each milestone to prevent role drift across disciplines and sites.

#### Defining Project Goals and Management

##### Choosing a Project Name

All consortium members should have the opportunity to propose ideas for a suitable project name. After gathering suggestions, potential candidates can be discussed within the team, culminating in a final vote and confirmation by the project manager. In our case, the name needed to represent both medical education and the integration of AI technology. As a result, we chose medical tr.AI.ning, emphasizing the acronym “AI” to highlight its dual focus and delivering a memorable, descriptive name.

##### Setting Project Goals and Milestones

Project goals and milestones must be clearly defined to ensure structured progress. Goals must align with the intended educational outcomes, such as improving procedural skills or enhancing clinical decision-making. Based on our experience, this step should precede detailed scenario design to ensure alignment between educational objectives and technical implementation.

Milestones should track key phases, including content development, technical implementation, user testing, and iterative evaluation cycles. Each milestone should be specific, measurable, achievable, relevant, and time-bound (SMART), including tasks such as completing scenario designs, integrating feedback from medical experts, and conducting beta testing. Regular reviews are essential to adjust for technical challenges or evolving educational requirements.

In the medical tr.AI.ning project, we identified 4 milestones (Textbox 1).

**Milestones of the medical tr.AI.ning project.**

**1. Developing a robust virtual reality foundation and authoring tool**

Creating realistic medical scenarios with a focus on artificial intelligence (AI)–supported visual elements; designing the core functions of the virtual reality (VR) environment and the authoring tool.

**2. Designing user navigation and smart interaction features**

Implementing navigation functions within the VR environment, a clear visual design, and intelligent interaction features.

**3. Enabling natural language and gesture-based interactions**

Leveraging AI-based speech interaction for input and output; additionally, developing an optional gesture-based interface.

**4. Integration and evaluation**

Integrating all developed components into a comprehensive, user-friendly VR application and conducting thorough evaluations; finalizing and using the authoring tool to create additional VR scenarios.

##### Developing a Project Timeline

The project timeline should follow a phased approach with clear, realistic deadlines. This typically begins with the discovery phase, which includes goal setting, stakeholder alignment, and requirements gathering. Next, the design phase should focus on content creation, scenario mapping, and visual asset development, followed by the development phase, which covers programming, VR integration, and interactivity design. The testing phase involves feedback from medical experts and beta testing [18].

Finally, the deployment phase ensures that the scenarios are refined and ready for educational use. Periodic reviews should be scheduled to assess progress and make adjustments as needed. After the VR scenarios are integrated into the curriculum, feedback from students should be collected to optimize both usability and content.

#### Budgeting and Allocation of Resources

##### Key Cost Components and Considerations

Budgeting and resource allocation should be based on well-defined objectives, with careful attention to relevant cost categories. Key cost components include personnel expenses for medical (didactical) experts, designers, and developers, which often constitute the largest share. Other substantial costs include licenses for software tools and assets (eg, VR development platforms, 3D modeling software, communication, and data storage tools). Hardware costs for head-mounted displays (HMDs), powerful computers, and necessary accessories (eg, cables, tripods, hygiene equipment) also represent a substantial portion of the budget.

Additionally, providing technical support to manage ongoing maintenance and supervise related courses is essential. In the medical tr.AI.ning project, this was done by research assistants. For dissemination, costs related to presenting the project at national and international conferences, publishing in open-access venues, and ensuring public accessibility (eg, through a dedicated website) should be included in the calculations.

Cross-departmental collaboration plays a critical role in ensuring that financial resources are appropriately aligned with project milestones. Regular reviews should be scheduled to adjust for evolving needs and unforeseen expenses, ensuring that the project remains on budget and achieves its objectives.

##### Securing Financial Support: Identifying Appropriate Project Calls and Tenders

Securing financial support is essential to account for the interdisciplinary nature of the consortium and the associated costs. Researchers should actively explore grant opportunities and funding programs focused on technology in education, health care innovation, and digital transformation. Priority should be given to funding calls that emphasize interdisciplinary research; medical training innovations; and VR, augmented reality, or extended reality technology. Funding sources can include national and international project calls, European Union programs such as Horizon Europe, or regional grants targeting medical technology. Partnerships with private-sector companies specializing in educational medical hardware or software should also be considered to supplement public funding and potentially facilitate subsequent commercialization.

Additionally, it is crucial to designate a team member or external consultant to take responsibility for securing funding for follow-up projects early in the development cycle. This ensures continuity of the research and development pipeline, allowing the consortium to build on its initial achievements and scale the project’s impact. Early identification of future funding opportunities and alignment with upcoming calls or partnerships can help avoid interruptions in development and support the long-term sustainability of the initiative.

The medical tr.AI.ning project was funded through the program “Artificial Intelligence in Higher Education” by the German Federal Ministry of Education and Research for a 3-year period (grant 16DHBKI077). The project was successfully submitted in the subcategory “national cooperative research project.”

#### Minimum Viable Requirements

Based on our experience, a minimal viable setup for implementing VR courses in medical education includes (1) a core team consisting of at least 1 didactical coordinator, 1 clinical content expert, and 1 technical lead; (2) standalone VR hardware with institutional device management capabilities; (3) a dedicated physical space that allows safe VR use; and (4) basic evaluation instruments assessing usability, acceptance, and perceived learning benefit.

#### Key Actions (Phase 1)

- Define the curricular gap and justify why VR adds value compared with existing methods.
- Translate the gap into competency-based learning objectives (eg, NKLM 2.0).
- Build the consortium and assign roles.
- Define SMART milestones and a phased timeline, including pilot testing and curricular integration.
- Decide on the minimum viable setup (hardware, room, staffing, hygiene, evaluation instruments).

### Phase 2: How to Organize

#### Overview

This phase addresses the organizational and managerial aspects of VR project implementation. It provides practical guidance on project management structures, task allocation, interdisciplinary communication, and technical infrastructure planning.

#### Communication and Data Storage

A key aspect of successful implementation is communication. Therefore, the respective platforms should be defined early, along with rules for notifications. Additionally, an efficient meeting schedule should be established, including agendas and time for discussing difficult topics.

Hybrid and remote team setups introduce unique challenges in maintaining communication, collaboration, and cohesion. Therefore, establishing clear rules for asynchronous and synchronous communication is vital to minimizing delays and misunderstandings. Team members should have access to well-defined workflows and tools tailored for remote use.

A minimal viable setup includes a shared cloud platform, a messaging tool for asynchronous communication, and a calendar system for scheduling regular review meetings. However, the abundance of available communication tools, such as email, communication and collaboration platforms (eg, Microsoft Teams, Slack, Mattermost), as well as video conferencing tools, can often lead to confusion if not properly managed. In hybrid or remote setups, balancing synchronous (eg, video calls) and asynchronous (eg, collaboration platforms) communication becomes essential to accommodate team members in different locations. Informal virtual spaces, such as casual chat channels, can help build team rapport and compensate for the lack of face-to-face interaction. In our project, we opted to use email for formal communications and important updates.

Meetings were scheduled, and calendar invites were shared via Microsoft Outlook, reducing the need for frequent reminder emails. Online meetings were conducted via the video conferencing tool Zoom (Zoom Communications, Inc), using a permanent access link that ensured easy retrieval and accessibility for all project members at any time.

For informal and real-time discussion, we used the collaboration platform Mattermost (Mattermost Inc), which was hosted by the Center for Information Technology, University of Münster. Several topic-specific channels were defined to keep conversations organized and prevent information overload. A reasonable allocation of members to channels helped prevent flooding with undirected information and kept discussions focused on relevant themes. However, the number of communication channels should be limited to avoid overwhelming team members with multiple sources of information.

A fixed meeting cadence supports transparency and alignment in interdisciplinary VR projects. Based on our experience, we recommend a 2-tier structure:

- Weekly working meeting (60-90 minutes): All research associates review progress, clarify blockers, and prioritize tasks. Meetings may alternate between development-focused discussions and milestone planning.
- Monthly consortium meeting:
  Project leads and working group representatives review overall progress, address strategic decisions, and confirm next steps.

Clear agendas and concise written minutes should document decisions and assigned responsibilities. The meeting structure should be adapted as the project transitions from exploration to implementation and curricular integration phases.

To share and store all relevant data, a common cloud solution should be used that complies with national data protection regulations. This is particularly important in the medical context when dealing with sensitive data (eg, patient or study data) used or collected during the project. It is also essential that all parties have equal access to the cloud and that its capacity is sufficient for software-based projects. In our case, we used the university cloud for North Rhine-Westphalia, “Sciebo” (version 5.3.1.14360; University of Münster, Münster, Germany).

Moreover, it is advisable to use a designated agile project management framework, for example, Scrum [19], and tools such as Kanban [20] to provide an overview of current tasks and ongoing work. The iterative approach of agile software development, with short development cycles, facilitates continuous feedback and improvement, which is especially beneficial in complex, interdisciplinary projects. Kanban serves as a visual workflow management method designed to optimize efficiency and productivity by tracking tasks on a board divided into columns, typically representing stages of a process.

In our project, we used the Boards function of the Mattermost platform as a Kanban tool for meeting agendas and protocols, as well as the ticketing function of GitLab (GitLab Inc) for specific work assignments in software development. In addition, we partially used selected methods of Scrum and agile software development for the creation of the VR simulation.

While short iteration cycles enabled early evaluation and adaptation of VR prototypes, the complexity of VR development often exceeded the constraints of short sprints. Therefore, we did not prioritize all aspects of the Scrum framework, although, in retrospect, stricter use of this method could have helped streamline some steps of the development process.

#### Lessons Learned and Implications for Future Implementations

If the project were to be implemented again, several structural decisions would be adjusted based on our experience. In particular, establishing a central technical coordinator from the outset would be prioritized to improve cross-site synchronization, reduce redundancy, and streamline technical decision-making.

In addition, a more consistent application of agile project management practices, including clearer sprint definitions, responsibilities, and review cycles, could further enhance development efficiency and transparency.

Finally, earlier and more systematic alignment of terminology and expectations across disciplines may help mitigate communication barriers inherent in interdisciplinary VR projects. These reflections highlight not only what proved effective but also which aspects require adaptation to improve future implementations.

#### Key Actions (Phase 2)

- Agree on 1 primary channel for asynchronous communication and 1 for formal decisions.
- Implement a shared task board (Kanban) and a source-of-truth repository for files.
- Set a fixed meeting cadence (weekly working meeting + monthly consortium steering).
- Define infrastructure responsibilities (room booking, device management, updates, and hygiene).

### Phase 3: How to Build

#### Overview

This phase focuses on the practical development of VR applications for medical education. It outlines design considerations, interaction concepts, and technical decisions that guide the implementation of educationally meaningful VR simulations.

#### Educational Context

##### Defining Learning Objectives

To ensure sustainability, the educational context of a VR-based medical education project should be closely aligned with current competency-based learning objective guidelines, such as those outlined in the German National Competency-Based Learning Objectives Catalogue for Medicine (NKLM 2.0 [17]). This alignment helps ensure that the VR simulations directly address the core competencies and skills expected of medical graduates and educators. Furthermore, alignment with established national and international medical education frameworks helps keep the content relevant, which ultimately fosters not only institutional support but also adoption by other medical schools.

In the medical tr.AI.ning project, we initially focused on dermatological education and defined the corresponding learning objectives based on the NKLM 2.0 before the development of the actual application. A strong overall focus was placed on the competency “clinical reasoning,” which refers to the process by which medical professionals gather and analyze patient information, generate differential diagnoses, and make informed decisions about patient management [21].

##### Discussions of Technical Feasibility

The requirements arising from the course design for the VR simulation must be discussed with the technical team members to assess their feasibility and, if necessary, revise the concept. This includes, for example, requirements for the environment, objects, and examination tools, as well as the behavior of virtual patients and the necessary interactions within the simulation.

##### Defining Course Structure

The course structure for VR-based courses in medical education should be designed to target specific, predefined learning objectives and requirements. A modular design approach divides the course into distinct sections, each focused on specific competencies, such as clinical procedures or clinical reasoning, allowing for independent updates to individual modules. A blended learning strategy combines immersive VR simulations with traditional lectures, workshops, or online materials, providing a comprehensive learning experience that supports both practical application and the necessary theoretical knowledge. As part of this strategy, theoretical input should precede the VR simulation to ensure that students have sufficient knowledge to complete the respective tasks in VR. Additionally, offering a VR tutorial session is advisable to help students become familiar with the navigation and controls.

Courses can either be self-paced, allowing students to learn at their own pace and offering flexibility for their schedules, or instructor-led, ensuring structured guidance and personalized feedback from experts. Scenario-based learning within VR allows students to engage in real-world medical situations, promoting critical thinking and decision-making under realistic conditions.

Finally, integrating assessment and feedback mechanisms, such as formative and summative evaluations, ensures continuous tracking of student progress, alignment with competency-based educational goals, and the individual feedback students need to continuously improve their performance.

**Figure 2:**
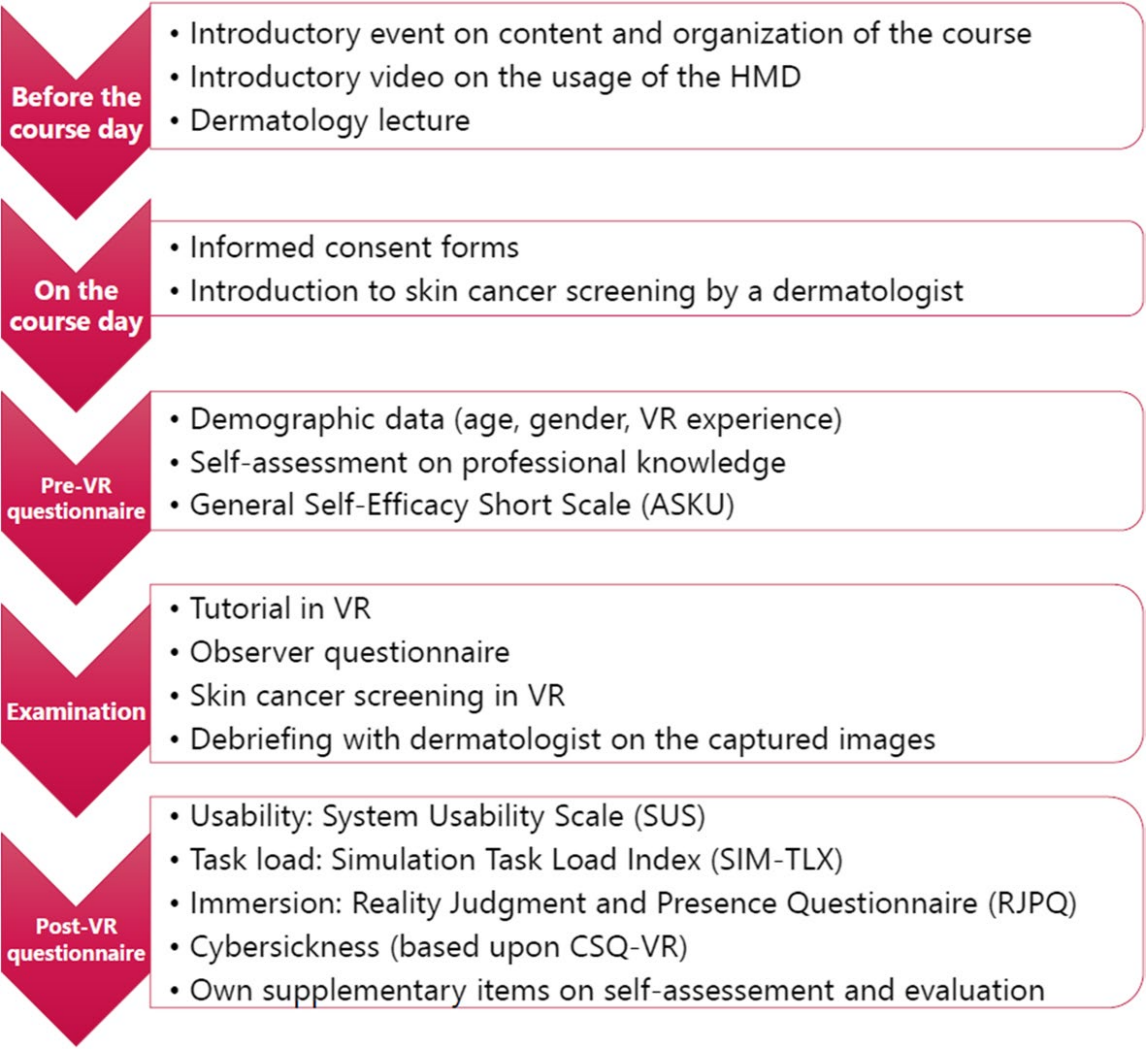
Time schedule and content of the virtual reality (VR) course in dermatology established at Saarland University [14]. HMD: head-mounted display. CSQ-VR: Cybersickness in Virtual Reality Questionnaire.

In the medical tr.AI.ning project, we started the course day by providing instructions and theoretical background knowledge on the strategy of a dermatological whole-body examination by a qualified dermatologist to ensure that students had sufficient theoretical knowledge to benefit adequately from practicing the procedure. After that, an interactive VR tutorial had to be completed, in which all necessary interactions could be practiced. Following the actual simulation, the results were discussed with the dermatologist, feedback was given, and questions could be asked. Figure 2 illustrates the overall structure and sequence of activities in the VR dermatology course (also see [14]).

#### Technical Foundation

##### Choosing VR Hardware

Several factors influence the decision regarding the most appropriate VR hardware. Performance requirements are crucial, as the hardware must support high-quality graphics and real-time interactions to create immersive and realistic medical scenarios.

Ease of use and comfort are also important, particularly for longer training sessions, so lightweight headsets with ergonomic designs are preferred.

Cost and scalability are major considerations, as the hardware must be affordable for institutions and scalable to accommodate multiple users simultaneously. Durability and ease of maintenance should be factored in, as the hardware will likely undergo frequent use, necessitating reliable performance and straightforward repair or replacement options. Mobility is another key consideration, that is, whether the VR setup will be room-based or portable, wire-free or PC-tethered, depending on the logistical possibilities and learning objectives. To meet hygiene standards, disinfection equipment should be used to clean the hardware between users, and single-use hygiene masks should be placed inside the HMD.

As graphic resolution plays a substantial role, not only in the context of cybersickness [22] but also in the accurate representation of medical conditions, the “Valve Index” by Valve was chosen for the medical tr.AI.ning project at both locations in 2022. The special Knuckles Controllers of the Valve Index were favored because of their finger-tracking capabilities, which allow the hands to be used as naturally as possible in medical scenarios. Although the VR hardware market is rapidly evolving, future compatibility and dissemination strategy should be considered when choosing the appropriate hardware. For example, in our case, relying on Steam’s Valve Index VR Kit made transfer to other faculties challenging because of Steam’s credit card–only acquisition policy.

##### Providing Appropriate Infrastructure

**Figure 3:**
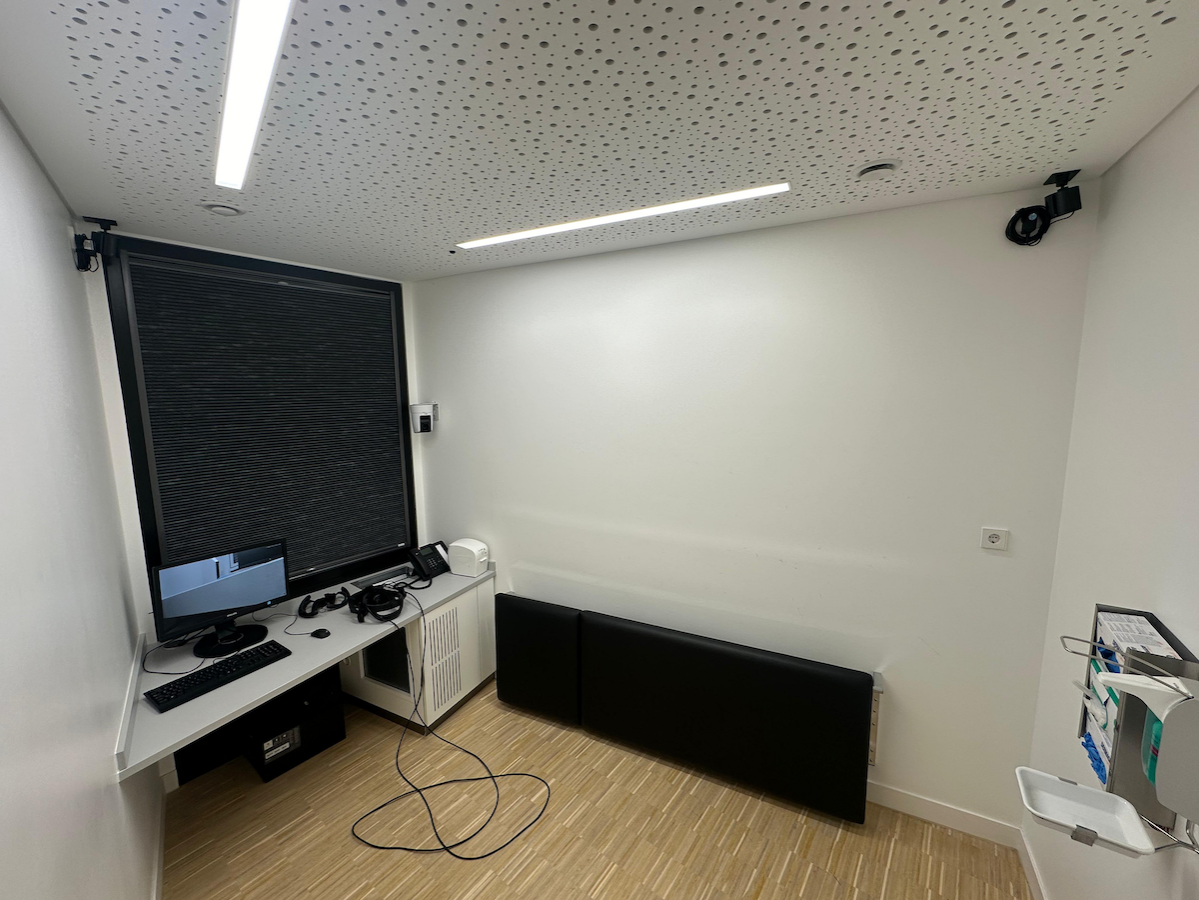
An equipped course room for a student at the Münster campus, located within the premises of the LIMETTE (Lernzentrum für individualisiertes medizinisches Tätigkeitstraining - Learning Center for Individualized Medical Skills Training) simulation training center, featuring technical upgrades to support the use of virtual reality hardware.

Ensuring appropriate infrastructure is crucial for the success of the project. The physical infrastructure should include dedicated rooms equipped with adequate space, power, and ventilation to accommodate VR hardware, such as HMDs, sensors, and powerful computers. These spaces need to be arranged to facilitate ease of movement and interaction (Figure 3).

On the technical side, IT support is essential to ensure that the VR hardware and software receive regular updates and troubleshooting when necessary. Network infrastructure is equally important, as VR simulations often require stable, high-bandwidth internet connections, particularly if cloud-based processing or collaborative simulations are involved. Additionally, the infrastructure should meet data management requirements, particularly if student performance data and feedback are being collected for evaluation or research purposes. This requires secure storage solutions that comply with data privacy standards, especially when handling sensitive information.

Finally, the infrastructure should be scalable to accommodate the growth of the project, whether through expansion to additional courses or by upgrading equipment as technology advances.

##### AI Frameworks, Methods, and Integration

###### AI for Interaction and Learner Support

Within the medical tr.AI.ning project, AI was explored as an enabling technology to support selected aspects of content creation and interaction design rather than as a central pedagogical driver of the VR course itself. At the time of project initiation in 2021, many AI-based approaches that are now widely available (eg, large language models [LLMs]) were still emerging or not yet sufficiently mature for reliable integration into medical education contexts. Consequently, several design decisions favored robust, manually implemented solutions over experimental AI-driven components to ensure clinical accuracy, usability, and curricular acceptance.

To facilitate realistic speech interaction with the virtual patients, the development of a specialized language interaction model was initially planned. To ensure that it would meet all requirements in terms of reliability and robustness, it was designed to build on established technologies and frameworks such as RASA (Really Awesome Software Automation) [23] to create a chatbot specifically adapted for use in medical scenarios. Together with AI-supported speech-to-text and speech synthesis models from Microsoft Azure, the system would have evolved into a complete dialogue system while remaining flexible and predictable at its core through the use of decision trees and scenario-specific intention and action systems for the chatbot.

However, with the initial public preview release of ChatGPT at the end of 2022, OpenAI caused a significant disruption in the research community focused on the development of intelligent speech-enabled agent systems. Consequently, our efforts to develop a novel, cutting-edge interaction model for virtual medical scenarios were suspended. The subsequent rapid developments and possibilities that emerged in the aftermath of the LLM surge quickly became apparent, prompting an expeditious transition to a robust menu-based interaction system in the project’s initial phase. Concurrently, the integration potential of novel LLM models was explored in small qualitative user studies.

Based on these findings, it became apparent that the models were not yet suitable for stable use in the medical curriculum. On the one hand, the additional overhead of the LLM caused the overall pipeline to be too slow to achieve a realistic conversational experience for the user. On the other hand, the model exhibited a tendency to frequently lose its role and hallucinate. Additionally, the capacity to effectively control tools was not yet sufficiently developed, which significantly hindered the synchronization of animations and actions. However, many of these issues have been mitigated or addressed in recent versions of LLMs. They currently offer great potential for the implementation of an intelligent speech-enabled agent system. A comprehensive evaluation within our system framework remains pending.

Considering the rapid development of AI technology, we recommend in-depth research on available tools and designing their integration in a flexible way to make short-term adjustments possible.

###### AI for Content Generation

AI also enabled us to explore new ways of generating content for learning applications that can easily be scaled up, allowing for more personalized learning experiences. We compared various image-generation techniques to create skin lesion images for our dermatological scenario. Ultimately, we opted to train our own diffusion model from scratch using publicly available data with real images [16]. We also added functionality to control the generation process, enabling us to create lesions directly on the virtual patient’s skin texture. This allowed us to create multiple scenarios with different lesions placed in various locations on the virtual patient’s body. For now, a medical expert has manually checked all of these lesions to ensure they are correct and do not contain unrealistic artifacts. However, it may be possible to fully automate the process in the future, providing an individual scenario for each student. Additionally, the generation process enables those preparing the course to customize the content to specific requirements. For example, we have also developed a tool that can generate lesions in a specific shape. This allows the focus to shift toward specific characteristics when identifying a malignant lesion, for example, more on texture than asymmetry.

Finally, the generated lesions can no longer be associated with a real patient, thereby helping to preserve patient privacy while still providing access to a nearly unlimited number of examples.

#### Interdisciplinary Communication

##### General Principles

Overall, interdisciplinary communication is a critical challenge that must be addressed effectively [24]. In addition to the typical difficulties faced by growing teams, there are further obstacles related to differing vocabularies and the potential for unrealistic expectations regarding the capabilities of other teams. Regular meetings are essential to quickly identify, uncover, and resolve misunderstandings. Additionally, visual representations of current development stages can help nontechnical team members stay aligned and informed.

##### Assigning Tasks

While this aspect may initially seem trivial, it is often overlooked in practice. Project tasks should be defined in great detail and assigned to specific individuals, making it clear who is responsible for each task. This reduces confusion and ensures that no critical aspect of the project is overlooked. It also facilitates better progress tracking, enabling project managers to identify potential delays or issues early on. Moreover, assigning tasks based on individuals’ skills or expertise ensures that the right people handle the right responsibilities, thereby improving the overall quality of the project. This structured approach promotes better collaboration, as each team member understands their role and contribution to the project’s success.

##### Defining Deadlines

Clear deadlines are essential for any project, as they provide structure, focus, and a sense of urgency, helping to keep the project on track. By setting deadlines, teams can prioritize tasks, allocate resources efficiently, and ensure steady progress toward milestones. In particular, for complex interdisciplinary projects, deadlines help synchronize the efforts of all disciplines, minimize bottlenecks, and ensure the timely delivery of the project.

In case of delays or when deadlines cannot be met, transparency with all cooperation partners is essential. Open communication helps to identify the underlying challenges and collaboratively develop solutions. One effective approach can be to reassign tasks to other team members or partners who are better positioned to complete them within the required time frame. This ensures that the project remains on schedule while maintaining quality.

#### Key Actions (Phase 3)

- Lock learning objectives and confirm technical feasibility with the development team.
- Select VR hardware based on visual fidelity, comfort, maintainability, and scalability.
- Establish an iterative build loop (prototype → medical expert review → user test → backlog).
- Decide whether AI is used for interaction support, content generation, or neither.
- Create a VR onboarding tutorial and define fallback options (cybersickness, accessibility).

### Phase 4: How to Integrate

#### Overview

This phase focuses on the systematic integration of VR applications into medical curricula. It addresses iterative testing, pilot studies, curricular embedding, dissemination strategies, and long-term sustainability.

#### Iterative Testing During Development: Small Iteration Evaluations With Medical (Didactic) Experts

Small iterative evaluations with medical (didactic) experts are essential to ensure the educational validity and content quality of VR simulations. Conducting these evaluations regularly throughout the development process helps identify potential issues early, ensuring that the content aligns with clinical practices, educational goals, and frameworks such as the NKLM 2.0. These evaluations can be organized as short, focused sessions at the end of each development iteration. In practice, we recommend proper documentation to ensure that feedback is manageable and directly actionable.

Especially, iterative testing and early collaboration with health care professionals provide essential expertise in defining appropriate real-world medical scenarios, procedures, and patient interactions that the simulations must replicate. Their input is critical for ensuring that the VR environments reflect the complexity, decision-making processes, and challenges faced in actual clinical practice. Additionally, medical experts help ensure that the simulations align with current medical standards, protocols, and guidelines, which is vital for the validity of the VR training content.

Alongside clinical accuracy, realism and user experience (UX) should also be regularly evaluated. Feedback is then incorporated into subsequent iterations, allowing for continuous refinement. In our project, we regularly tested simulation prototypes with board-certified dermatologists to ensure the accuracy and medical validity of the simulation. Feedback was documented and directly influenced the next development steps.

#### Pilot Studies: Testing the Prototype in Small User Evaluations

Testing the prototype in small user evaluations ensures that the product is user-friendly and aligned with the intended learning objectives. Small-scale testing allows for early detection of usability issues, such as interface design flaws, technical glitches, or difficulties in navigating within the VR environment. It also provides valuable insights into the simulation’s effectiveness in facilitating learning and whether students can successfully engage with the content to develop the targeted skills or knowledge. To conduct these evaluations, a small group of representative users, such as medical students or clinicians, should be selected to interact with the prototype.

Structured user testing sessions can be organized in which participants complete specific tasks within the VR simulation under observation. Based on our experience, this step should precede larger-scale deployment and ideally involve 5-10 representative students to capture diverse interactions with the VR environment. Feedback can be collected through postsession interviews, surveys, or direct observation of user interactions.

Quantitative metrics such as task completion time and error rates, along with qualitative feedback on ease of use and learning experience, should be gathered and analyzed. This iterative testing process helps identify areas for improvement before larger-scale deployment, ensuring that the final product is both effective and intuitive for its target audience.

Table 2 presents an overview of the evaluation activities carried out within the medical tr.AI.ning project that informed this tutorial, feasibility evaluation, and scientific contributions, as detailed below.

**Table 2:** Overview of evaluation activities informing this tutorial (medical tr.AI.ning, 2021-2024).

Evaluation type	Participants	Purpose/decision informed	Timing/setting
Feasibility study/curricular pilot	n=140 medical students	Technical feasibility + usability/presence/task load + perceived competence across teaching unit	Summer term 2023; University of Münster
Feasibility study/curricular pilot	n=58 medical students	Feasibility + usability/immersion/task load/cybersickness + perceived competence	Summer term 2023; Saarland University (Homburg)
Iterative SME^a^ reviews (dermatology)	3-5 board-certified dermatologists	Clinical accuracy checks; scenario realism; and checklist completeness	Throughout development iterations (2022-2024)
Authoring tool workshops/usability tests (educators)	5-7 medical educators	Requirements + user interface comprehension + task success; and refinement of authoring workflow	Iterative workshops/interviews (2022-2024)

^a^SME: subject matter expert.

In our project, it was important that medical teachers could create their own VR training scenarios, even without prior VR knowledge, by using a dedicated authoring tool. Therefore, in an iterative process, the UX designers gathered information about the teachers’ wishes and needs through workshops and interviews, leading to a user-centered design approach. Initial functional prototypes were repeatedly tested by the teachers to determine whether the functions were easily understood. The interface was developed through progressively refined iterations to ensure good usability and UX, and to help teachers identify with the authoring tool and gain the confidence to create their own VR scenarios.

Throughout the project, we collected both qualitative data (comments, opinions, and suggestions for improvement) and quantitative data (time spent on tasks, error rates, and use of specific functions) to verify design decisions and tailor the application to specific user requirements in an iterative process.

#### Integration Into the Curriculum

##### Integration and Organization of the VR Course

Implementing VR-based courses in medical education requires meticulous planning to effectively address practical aspects. Supervision during the course, such as by student assistants, must be well coordinated, with clearly assigned roles for overseeing student interactions within the VR environment and addressing potential technical issues. Access to dedicated rooms and facilities equipped with VR hardware, including HMDs and computers, is essential, and scheduling these resources must be streamlined to avoid bottlenecks. Regular hardware and software maintenance, including timely updates and technical support, is critical to ensuring smooth operation during sessions.

Both staff and students should receive an introduction to the course design, including training on VR tools and how the simulations align with learning objectives. To enhance the scientific value of the project and promote continuous improvement, we recommend that the project be scientifically monitored through evaluation methods. This involves data collection, such as automatic logging of performance data, user feedback, and assessment of learning outcomes. Where appropriate and with prior consent, additional measures such as external assessments or video recordings can also be used. Finally, depending on the hardware used, it must be ensured that all wireless components are fully charged before sessions to allow uninterrupted use.

Depending on the course format and its learning objectives, it may be advisable to provide alternative learning options for students experiencing cybersickness, allowing everyone to achieve creditable learning outcomes regardless of constraints. This comprehensive organization ensures the smooth integration of VR simulations into the medical education curriculum while fostering effective operation and alignment with institutional settings.

##### VR Tutorial

A VR tutorial should guide users in learning to interact with the software. The structure of the tutorial should ensure that students learn basic interactions at the beginning, such as teleporting and basic object interactions, before introducing more specific functionalities, such as using tools like the digital dermatoscope in the medical tr.AI.ning application.

Interactions should be demonstrated step-by-step, allowing students to follow along and understand the controls. Later, students should be asked to apply their knowledge without specific instructions. For example, navigation should be among the first skills introduced: students should learn how to move around the virtual environment early in the tutorial. Subsequent tasks should require them to navigate without further guidance, reinforcing their understanding of movement controls through active use. This progression from guided learning to independent application ensures that, by the end of the tutorial, students have a solid grasp of the necessary VR controls and can confidently apply them during the actual simulation scenarios. In the medical tr.AI.ning tutorial, students start in a doctor’s office and are familiarized with the environment. Gestures and functions to be learned are introduced within a medical context so that the subsequent transition to the actual medical examination simulation feels logical and intuitive.

##### Extensive Feasibility Evaluation

Establishing a clear assessment strategy is crucial to evaluating the effectiveness and acceptance of VR-based courses in medical education. This strategy should incorporate both formative and summative evaluations to measure acceptance among educators and students, as well as specific learning outcomes. In the initial stages, conducting extensive feasibility evaluations is particularly important. These evaluations should address key aspects such as usability, immersion, presence, cybersickness, and subjective learning outcomes. The findings from these studies provide valuable insights into the UX and educational impact, allowing for the refinement of subsequent prototypes. The respective evaluations of the medical tr.AI.ning project, based on the first courses conducted, have been published and are accessible for reference [14,25], providing detailed insights into the feasibility and effectiveness of the approach. In summary, both studies demonstrate technical feasibility and positive learner reception. Students reported high usability and immersion, alongside significantly increased self-assessed competence following VR training. The VR implementations were positively evaluated for their educational value while also drawing attention to resource and cost considerations relevant to broader curricular adoption.

By integrating these assessment practices, the project can ensure that the VR simulations align with educational goals, meet user expectations, and continuously improve based on empirical feedback.

#### Dissemination

##### Public Appearance

To ensure a consistent and unified visual identity for external correspondence, a logo should be developed to represent the project. The creation of a logo requires careful consideration and respect for the values of the individual working groups within the consortium. An iterative design process, with multiple review cycles, ensures that team members feel a sense of pride and unity in the final result. The logo should strike a balance between abstraction and inclusivity to fairly represent the diverse ideas and values contributed by all project partners. In addition, a project website should be developed to support the project’s public presence and facilitate contact with potential cooperation partners. Furthermore, representation at relevant conferences ensures visibility, provides opportunities for hands-on experiences with the developed simulations, and opens possibilities for future scientific collaborations.

The logo for the medical tr.AI.ning project was developed by designers within our consortium. It integrates multiple concepts into a cohesive design. The outer shape resembles a stylized brain, symbolizing the role of AI in the project. The sharp angles create the impression of a 3D ribbon, reflecting the VR application’s immersive and realistic content. Additionally, the line’s end points suggest the head and tail of a coiled snake, evoking the image of the Aesculapian staff, a symbol widely associated with medicine. This thoughtful integration of concepts underscores the project’s interdisciplinary nature and its connection to medical education. Emphasizing the use of AI, the letters “AI” were highlighted in the project name. The green color of the logo was adopted as the official project color to create a unified visual identity. In addition to the logo and colors, it is important that the interaction design and interface of both the authoring tool and the VR environment be developed in a consistent style. This approach reinforces the perception of unity, allowing teachers and students to intuitively recognize all elements as part of a single, integrated system.

To further enhance visibility and outreach, a dedicated project website was developed [26]. The website features a video-based project overview, illustrative images, and continuously updated content, such as details on congress participation, publications, and reports from project meetings. The website reflects the essential stylistic elements of the authoring tool and VR environment interface to ensure a clear association with the project.

To convey professionalism and quality at conferences, the designers created branded materials, including business cards, leaflets, and a booth adorned with posters. These were complemented by a hands-on setup of the VR simulation, allowing attendees to directly experience the application. During public testing rounds, close attention was paid to immediate user feedback. Such settings often bring together experts in medical education and VR technology, providing valuable recommendations and opening opportunities for potential collaborations.

The project was showcased through lectures, exhibition stands, and workshops at various national and international congresses. Notable events included “Würtual Reality” in Würzburg, “Shift Medical” in Heidelberg, the German-speaking countries (Germany, Austria, and Switzerland; DACH region) “Annual Conference of the Association for Medical Education” (GMA) in Osnabrück, and the “International Association for Health Professions Education Conference” in Basel, Switzerland.

##### Networking Groups

To support sustainability and foster collaborations, consortium members should actively participate in specialized working groups. In our case, we prioritized networking through the VR Working Group of the GMA, taking on both active participation and leadership roles. This working group facilitates regular exchanges of knowledge and resources, providing updates on relevant congresses, publications, and other critical developments related to VR in medical education. The medical tr.AI.ning project was presented within this context, significantly enhancing its visibility and recognition among professional circles across the DACH region. This exposure sparked interest from several universities in adopting the project following the completion of software development. Furthermore, it paved the way for potential collaborations exploring the integration of VR technology in medical education.

##### Sustainability

To ensure the value and usability of the developed project outcomes beyond the project’s life cycle, it is crucial to establish a distribution solution early in the process. A significant challenge in this endeavor is ensuring the transferability of the interface to various hardware systems. For example, in our case, the currently utilized finger-tracking functionality of Valve’s Knuckles Controllers enables interaction with objects using natural grasping movements. However, this feature is not supported by most other current controller variants, necessitating the development of a fully button-based alternative to maintain compatibility across different systems.

Additionally, legal and formal considerations must be addressed to provide clear guarantees for all stakeholders, including both developers and end users. This involves reviewing each asset used in the project to identify potential licensing conflicts. Where such issues arise, suitable alternatives should be identified and implemented to ensure the legality and accessibility of the final product.

Providing a dedicated authoring tool, medical asset library, and open-source solutions can enable developers and medical educators to build upon the work of the VR project.

##### Scientific Contributions

To provide value to the scientific community and other developers, it is essential to disseminate collected data and research findings through online articles and open-access publications. In our project, several open-access articles have been published, contributing valuable insights into the integration of VR in medical education. These publications address various aspects of the project, including its overall objectives [8], students’ perceptions and desires regarding the integration of VR into medical curricula [27], a scoping review of the current state of VR integration in medical education [1], and specific findings from implementing a VR simulation for skin cancer screening in the dermatology curriculum at both clinical sites involved in the project [14,25]. Collectively, these works offer a comprehensive perspective on the project’s potential and the evolving role of VR in advancing medical training, serving as a resource for future research and development in this field.

#### Key Actions (Phase 4)

- Run small usability tests (5-10 learners) before scaling to curricular pilots.
- Embed VR with clear staffing, room logistics, device cleaning, and technical support.
- Use a predefined evaluation set (usability, presence, task load, cybersickness, and learning perception).
- Plan dissemination (website, conferences, and publications) and address licensing early.
- Define postproject ownership: maintenance, hardware strategy, and scenario authoring workflow.

### Limitations

Several limitations should be considered when interpreting the scope of this work, particularly regarding its context-specific and transferable elements. First, the article follows a mixed format, combining aspects of a project report and a practice-oriented guideline. Its primary aim is to describe the development process of a multidisciplinary VR project rather than to evaluate educational outcomes. Accordingly, the work is predominantly descriptive, and conclusions regarding learning effectiveness or superiority over other teaching formats cannot be drawn from this paper.

The project was developed and implemented at 2 university sites (Münster and Saarland), and certain aspects are therefore context-specific. These include local infrastructural conditions, availability of VR hardware, existing technical expertise, and established interdisciplinary networks. Such factors influenced the pace and organization of the development process and may differ across institutions.

At the same time, the overarching project structure, development workflow, and interdisciplinary coordination strategies described in this paper are not tied to a specific institutional setting and may be transferable to other academic environments with comparable resources. The VR application itself is specific to dermatological skin cancer screening, a clinical domain that benefits from high visual fidelity and pattern recognition. Consequently, the developed assets and scenarios are not directly transferable to other medical disciplines. However, the underlying technical framework, including the modular software design and the AI-based generation of synthetic patient data to address data protection concerns, represents a transferable concept that may be adapted to other visually oriented medical fields, albeit with appropriate domain-specific modifications.

Resource requirements represent another important consideration. While the initial development phase was associated with substantial investments in time, coordination, and technical setup, the resulting course format can be implemented with comparatively moderate ongoing effort. Institutions without existing VR infrastructure or access to external funding may face higher entry barriers, particularly if limited hardware availability restricts scalability. Nevertheless, these constraints primarily affect project initiation rather than the applicability of the general development principles outlined in this article.

From a technical standpoint, development and testing were conducted using a single VR system (Valve Index). Although the software was designed with open-source compatibility and platform independence in mind, UX and performance may vary across different hardware environments. Finally, while the interdisciplinary collaboration constituted a key strength of the project, differences in disciplinary terminology and perspectives occasionally required additional coordination efforts. The final VR scenario focuses on a single-player setting and therefore only partially represents interpersonal and nonverbal aspects of clinical encounters, which should be considered when interpreting the scope of addressed competencies.

### Conclusions

This tutorial translates the 3-year, multiinstitutional implementation experience of medical tr.AI.ning into a practical, phase-based workflow for educators and developers who want to establish VR in medical curricula. Across the 4 phases detailed in this tutorial, the central message is that successful VR adoption depends less on technology alone than on a clear educational rationale, interdisciplinary coordination, iterative feedback loops, and early planning for sustainability and dissemination.

The following conclusions summarize the key steps and recommendations of this tutorial and synthesize the central lessons learned from developing and integrating VR courses in medical education (Textbox 2).

**Key steps and recommendations.**

**1. Identify and justify educational needs**

Before integrating a virtual reality (VR) course into the medical curriculum, conduct a comprehensive needs assessment to identify specific gaps in the curriculum. Ensure that VR adds unique value by addressing these gaps and improving the curriculum, rather than simply replacing existing teaching methods.

**2. Assemble an interdisciplinary team**

Form a diverse team with expertise in medical education, computer science, 3D design, user experience/user interface design, and VR development. Clearly define roles, including project managers, didactic coordinators, and technical coordinators, to streamline responsibilities and collaboration.

**3. Define clear project goals and milestones**

Set specific, measurable goals and milestones aligned with the desired educational outcomes early on, such as improving procedural skills or clinical decision-making. Establish clear deadlines to track progress effectively.

**4. Use a structured project management framework**

Implement a project management framework to organize development tasks, enable regular feedback loops, and ensure iterative progress throughout the project life cycle.

**5. Align VR content with competency-based learning objectives**

Design VR simulations to align with competency-based educational guidelines, ensuring relevance and standardization. This alignment ensures that the simulations directly support the desired learning outcomes.

**6. Conduct iterative testing and evaluation**

Perform regular, small-scale evaluations with medical, didactic, and user experience experts during development. This iterative approach ensures clinical accuracy, pedagogical effectiveness, and usability before broader deployment.

**7. Choose appropriate VR hardware and infrastructure**

Select VR hardware that meets the project’s performance, comfort, and scalability needs. Ensure the infrastructure supports reliable use, including dedicated space, stable network connectivity, and regular maintenance protocols.

**8. Organize efficient communication and data management**

Establish clear communication channels integrated with project management tools. Utilize a central platform for updates, task tracking, and data sharing to avoid confusion and ensure a consistent information flow among team members.

**9. Integrate VR into the curriculum thoughtfully**

Plan for the seamless integration of VR into the existing curriculum early on, and train all educators and assistants to feel comfortable with the technology. Technical support should be readily available.

**10. Plan for long-term sustainability and dissemination**

Address sustainability early, including transferability to other hardware and legal aspects such as licensing. Promote the project through a professional website, conferences, and open-access publications to ensure visibility and encourage adoption by other institutions.

## Abbreviations

AI: artificial intelligence
CHELM: Coordination Center Homburg for Education & Learning in Medicine
CVMLS: Computer Vision & Machine Learning Systems
DACH: German-speaking countries (Germany, Austria, and Switzerland)
ETI: Department of Electrical Engineering and Computer Science
FHMS: University of Applied Sciences Münster
GMA: Association for Medical Education
GUD: Institut Gesellschaft und Digitales
HBKsaar: Hochschule der Bildenden Künste Saar
HMD: head-mounted display
IfAS: Institut für Ausbildung und Studienangelegenheiten
LLM: large language model
MSD: Münster School of Design
NKLM: German National Competency-Based Learning Objectives Catalogue for Medicine
RACI: Responsible, Accountable, Consulted, Informed
RASA: Really Awesome Software Automation
SMART: specific, measurable, achievable, relevant, and time-bound
UI: user interface
UMTL: Ubiquitous Media Technologies Lab
UX: user experience
VR: virtual reality
